# Recent and future trends in sea surface temperature across the Persian Gulf and Gulf of Oman

**DOI:** 10.1371/journal.pone.0212790

**Published:** 2019-02-28

**Authors:** Roohollah Noori, Fuqiang Tian, Ronny Berndtsson, Mahmud Reza Abbasi, Mohammadreza Vesali Naseh, Anahita Modabberi, Ali Soltani, Bjørn Kløve

**Affiliations:** 1 School of Environmental, College of Engineering, University of Tehran, Tehran, Iran; 2 Department of Hydraulic Engineering, State Key Laboratory of Hydroscience and Engineering, Tsinghua University, Beijing, China; 3 Department of Water Resources Engineering & Center for Middle Eastern Studies, Lund University, Lund, Sweden; 4 National Institute of Oceanography and Atmospheric Science, Tehran, Iran; 5 Department of Civil Engineering, Faculty of Engineering, Arak University, Arak, Iran; 6 Water Resources and Environmental Engineering Research Unit, Faculty of Technology, University of Oulu, Oulu, Finland; Universidade de Aveiro, PORTUGAL

## Abstract

Climate change’s effect on sea surface temperature (SST) at the regional scale vary due to driving forces that include potential changes in ocean circulation and internal climate variability, ice cover, thermal stability, and ocean mixing layer depth. For a better understanding of future effects, it is important to analyze historical changes in SST at regional scales and test prediction techniques. In this study, the variation in SST across the Persian Gulf and Gulf of Oman (PG&GO) during the past four decades was analyzed and predicted to the end of 21^st^ century using a proper orthogonal decomposition (POD) model. As input, daily optimum interpolation SST anomaly (DOISSTA) data, available from the National Oceanic and Atmospheric Administration of the United States, were used. Descriptive analyses and POD results demonstrated a gradually increasing trend in DOISSTA in the PG&GO over the past four decades. The spatial distribution of DOISSTA indicated: (1) that shallow parts of the Persian Gulf have experienced minimum and maximum values of DOISSTA and (2) high variability in DOISSTA in shallow parts of the Persian Gulf, including some parts of southern and northwestern coasts. Prediction of future SST using the POD model revealed the highest warming during summer in the entire PG&GO by 2100 and the lowest warming during fall and winter in the Persian Gulf and Gulf of Oman, respectively. The model indicated that monthly SST in the Persian Gulf may increase by up to 4.3 °C in August by the turn of the century. Similarly, mean annual changes in SST across the PG&GO may increase by about 2.2 °C by 2100.

## Introduction

Sea surface temperature (SST) variations under climate change influence species in the marine environment and may thus threaten sensitive ocean corals, alter the intensity and frequency of blooms, reduce the nutrient flux from the deep to surface waters, raise sea level, change the global food chain, and create health-related problems for humanity by providing a more suitable environment for pathogenic microbes [1–6]. The variations in SST can also have a significant impact on climate components [7–10]. Several studies have demonstrated that SST has increased at global scale during the 20^th^ century [11–13], while regional studies in some parts of the world have found that shallow waters such as gulfs may display a larger variation in SST increase compared with deep water areas [14]. This may be a result of the relatively lower water depth in shallow water bodies [15–17]. Gulfs are mainly affected by their surrounding land mass and therefore the SST in such water bodies is also affected by air temperature, which consequently leads to more variability. For example, the decadal rate of SST increase in Narragansett Bay, USA, which is about 1.1 °C, is four times greater than that of the main ocean [18].

The Persian Gulf and Gulf of Oman (PG&GO) are important water bodies from an economic, political, environmental, and social perspective. Sea surface temperature in PG&GO has shown an increasing trend during recent decades [19–25]. However, climate change effects on SST variations are not similar in either time or space and, although reported results reveal an increasing trend in global mean SST, data on the trends at regional level are limited and sometimes inconsistent [8,26,27]. In addition, climate change effects on future trends and variations in SST will be different depending on region, due to driving forces including potential change in ocean circulation and internal climate variability, ice cover, thermal stability, and ocean mixing layer depth [28]. Therefore, far-reaching analysis at regional scale using measured SST and prediction of future SST would help authorities to mitigate harmful effects of global warming on marine ecosystems. In particular, long-term prediction of SST is important at regional scale for PG&GO, due to local effects on marine ecosystems, local climate, and beyond. Researchers have attempted to project future SST using different types of global circulation models [29–31]. However, these models are complex and consist of different components such as atmosphere, ocean, land, sea ice, chemistry, and biology [32]. This makes them data heavy and they require large amounts of measured data in order to make reliable predictions. A simpler model may be relevant when data availability is sparse.

The aim of this study was to evaluate spatiotemporal variations in SST across PG&GO, which is located in the Middle East between Iran and the Arabian Peninsula. For this purpose, variations in daily optimum interpolated SST anomaly (DOISSTA) data for PG&GO during the past four decades, which are available from the National Oceanic and Atmospheric Administration (NOAA) in the United States, were analyzed. The analysis comprised two stages. In the first stage, spatiotemporal descriptive statistics on the DOISSTA data were investigated for PG&GO. In the second stage, the proper orthogonal decomposition (POD) method was used to capture dominant modes of DOISSTA. The POD model have previously been shown to represent accurately the pattern of DOISSTA variation in the northeastern part of the Indian Ocean [33]. In addition, SST across PG&GO during the 21^st^ century was predicted using the POD model, which learns potential future warming patterns from the past behavior of historical DOISSTA trends. The POD approach has been widely applied in the field of computational fluid dynamics, but its application has been more rarely reported in oceanic and atmospheric studies [34,35].

## Study area

**Fig 1:**
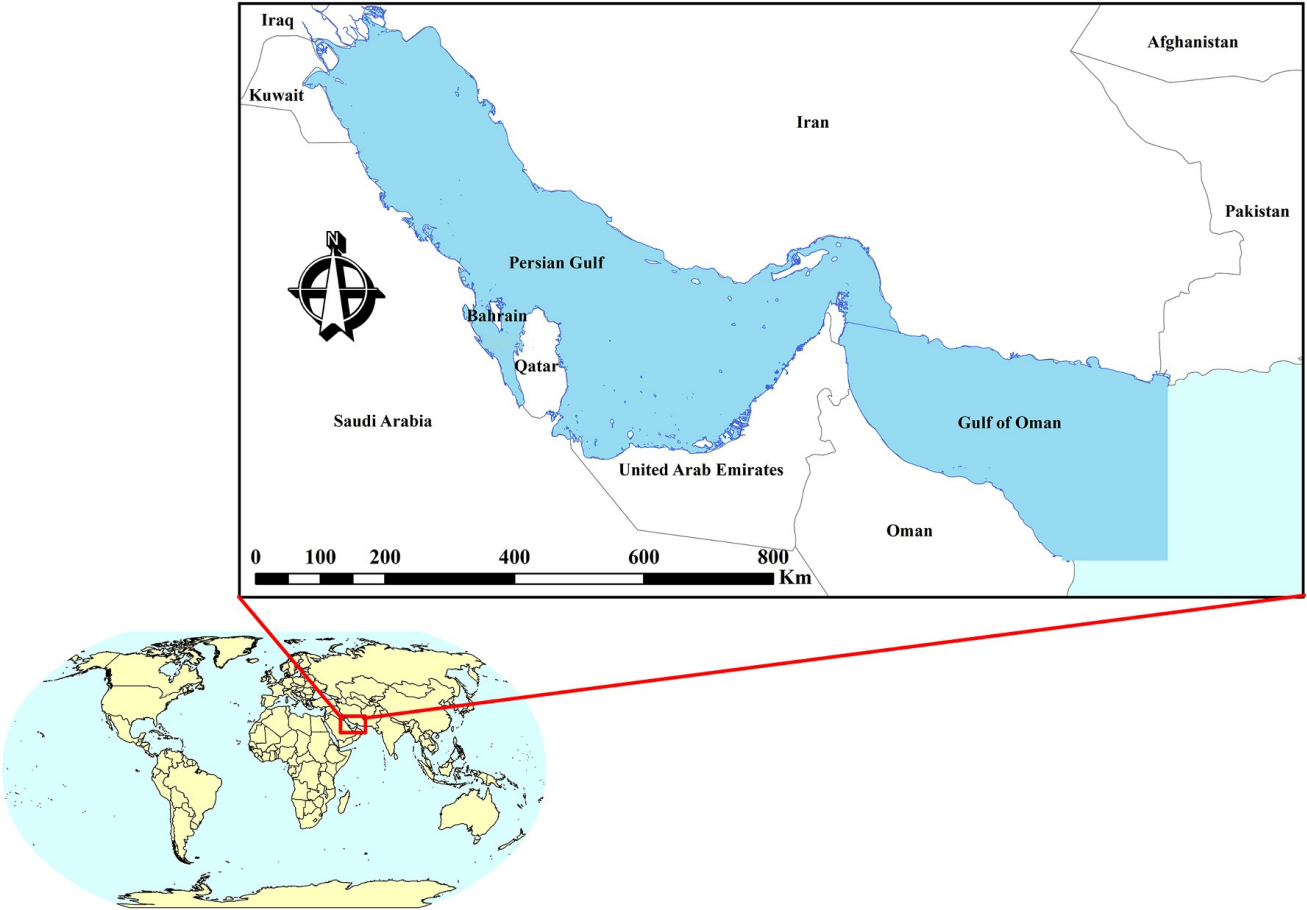
Map showing the study area and surrounding countries.

The Persian Gulf and Gulf of Oman, a climate change hotspot [36], borders Iran in the north, Iraq and Pakistan in the northwest and northeast, respectively, and Oman, United Arab Emirates, Qatar, Kuwait, Bahrain, and Saudi Arabia in the south (Fig 1). The water mass is connected to the Arabian Sea and ocean water in the east. The climate of surrounding land is dry and subtropical. Air temperature exceeds 50 °C in summer and evaporation rate exceeds rainfall in PG&GO. Mean annual rainfall along the southern and northern coasts is less than 50 and 200 mm, respectively. Freshwater discharge into PG&GO is mainly provided by the rivers of Iran, among which Arvand Rud provides the largest share. On the south coast, small amounts of freshwater flow into PG&GO. Higher salinity concentration in PG&GO than in the Indian Ocean, especially in western parts including the Persian Gulf, is the main factor influencing water exchange between PG&GO and the ocean [37,38].

The study area consists of the largest and most important transportation network between Europe, Africa, and South Asia, and it includes two-thirds and one-third of the world’s known oil and gas reserves, respectively [39]. Moreover, the water body is one of the largest habitats for marine organisms, such as corals, fish, and mammals [19,20,40,41]. Considering the importance of PG&GO, environmental and ecosystem protection has always been a challenge for the surrounding countries. Oil platforms and refineries around the Persian Gulf, discharge of oil, chemicals, and waste into the water, oil and gas transport in huge tankers, and outbreak of several wars in this region are the main factors threatening aquatic ecosystems of PG&GO [19]. This, together with the impact of climate change on aquatic ecosystems of PG&GO, have caused unsustainable conditions in the water body. A SST increase could exacerbate the effects of different pollution loads in PG&GO [42].

These challenges have prompted the coastal states of PG&GO to pay particular attention to this water body during implementation of national development programs, so as to protect the future sustainability of PG&GO. To assess the success of this work, this study investigated recent and future trends in SST across PG&GO.

## Materials and methods

Previous studies have investigated SST variations using monthly data [43,44], while Shaltout and Omstedt [45], Hobday et al. [46], and Noori et al. [33] used daily SST data to study marine extreme temperatures. Generally speaking, the variability is higher for daily SST data than for data recorded at monthly time scales, reaching about 1.5 times higher in some parts of the ocean [28]. As monthly SST datasets are averaged from daily data, they are smoothed and do not properly show extreme high and lows of SST. Therefore, in this study we used DOISSTA data to reveal the high and low extremes of SST across PG&GO. The DOISTA data available from the NOAA website have a spatial resolution of 0.25 degree on a daily basis. The data comprise multiple sensor observations (satellites, ships, and marine buoys). More details on the DOISSTA database are presented in Reynolds et al. [47] and Reynolds [48].

**Fig 2:**
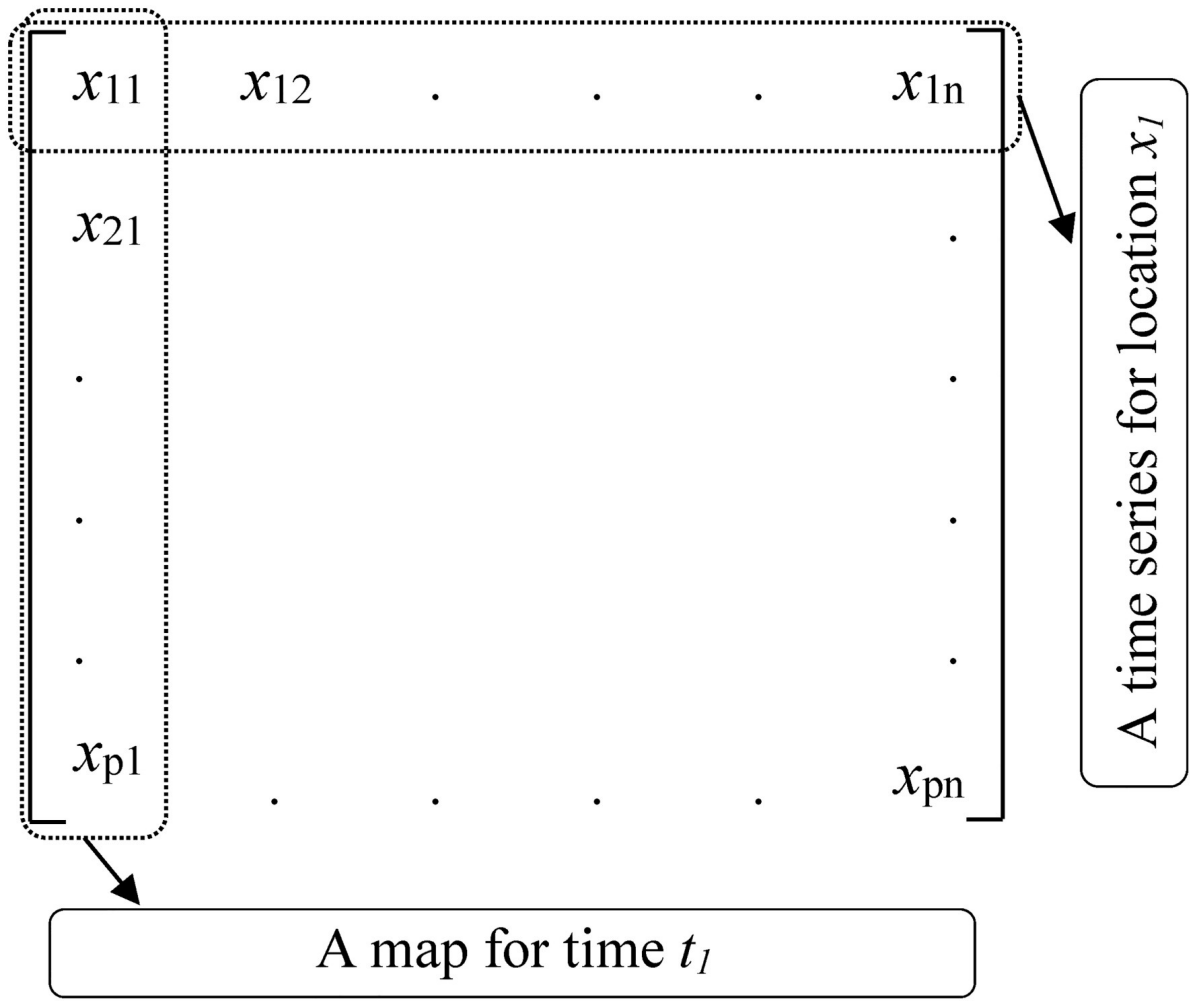
Prepared daily optimum interpolation SST anomaly (DOISSTA) data in matrix format.

The DOISSTA database covers the period from January 1982 to December 2015. The data are available in NETCDF format and can be loaded and managed by the MATLAB software. The extracted DOISSTA data for our study area resulted in 609 square 0.25 degree grids, each of which contained DOISSTA data for a total of 12,784 days. Thus, the final database was a matrix for DOISSTA containing 609 rows and 12,784 columns, as shown in Fig 2. In the matrix, rows indicate the number of grids (*x*_*i*_) and columns represent the number of days (*t*_*i*_).

**Fig 3:**
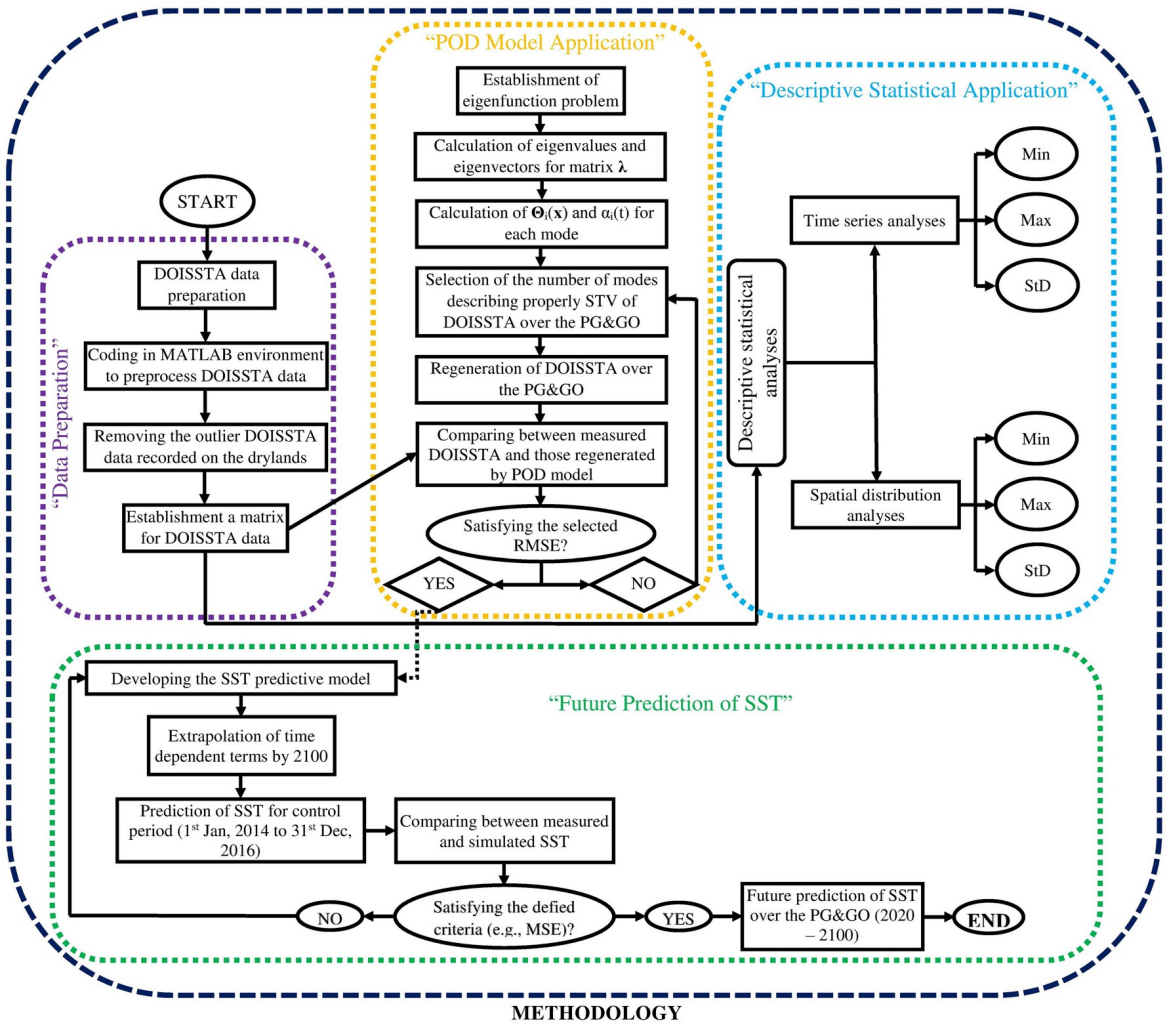
Schematic diagram of the analytical procedure involving the POD method used in the present study.

The DOISSTA matrix data were first analyzed in terms of descriptive statistics such as mean, minimum, maximum, and standard deviation (Fig 3). In the second stage, the POD method was used to extract the dominant spatiotemporal patterns in DOISSTA for the study area (Fig 3). The POD method was independently introduced by a number of scientists, e.g., Kosambi [49], Loeve [50], Karhunen [51], Pougachev [52], and Obukhov [53]. It is a statistics-based method that extracts spatiotemporal structures of an objective parameter available from simulations or experiments [33]. In other words, POD is a linear procedure for extracting a basis for modal decomposition from an ensemble of data called “snapshots”. It is noteworthy that, although the POD is a linear procedure, it is as blind as Fourier analysis and makes no assumptions on the linearity of the objective parameter [54]. However, the main advantage of POD lies in its mathematical properties, which render the method the preferred basis to use in many situations [54]. By application of the POD method, low-dimensional approximations of a high-dimensional problem can be optimally obtained. Therefore, the POD always results in a finite number of modes that properly capture the dominant features of an infinite objective problem [55].

When applying POD to the DOISSTA data, spatial and temporal components (**Θ**_*i*_(**x**) and *α*_*i*_(*t*), respectively) were considered according to [56,57]:

$$DOISSTA(x,t)=\sum_{i=1}^{N}\alpha_{i}(t)\Theta_{i}(x)$$ (1)

In order to calculate **Θ**_*i*_(**x**), it is necessary to solve Eq (2) to estimate the eigenvalues and their corresponding eigenvectors [58,59]:

|1−λI|=0 (2)

where λ is the eigenvalue such that *λ*_1_ ≥ *λ*_2_ ≥ *λ*_3_ ≥ ⋯ ≥ *λ*_*N*_ ≥ 0 and **I** is the unit matrix. Having eigenvalues and their corresponding eigenvectors, one can calculate **Θ**_*i*_(**x**) using Eq (3) [60,61]:

$$\Theta_{1}(x)=\sum_{i=1}^{N}\beta_{1i}DOISSTA^{\left(i\right)},\Theta_{2}(x)=\sum_{i=1}^{N}\beta_{2i}DOISSTA^{\left(i\right)},\ldots ,\Theta_{N}(x)=\sum_{i=1}^{N}\beta_{Ni}DOISSTA^{\left(i\right)}$$ (3)

where *β*_*ii*_ are eigenvectors corresponding to the eigenvalues. Finally, *α*_*i*_(*t*) can be calculated by [62,63]:

α(t)=(DOISSTA(x,t),Θ(x)) (4)

In the POD application, the first few eigenvalues are most important, as they represent the main energy in the system (here DOISSTA variations in PG&GO) [64]. Thus, by using the first few modes (*K*) corresponding to the first few calculated eigenvalues, it was possible to represent DOISSTA variations in PG&GO. In addition, using just the first few modes one can regenerate DOISSTA data using Eq (5) [56].

$$DOISSTA(x,t)≅\sum_{i=1}^{K}\alpha_{i}(t)\Theta_{i}(x)\;K\ll N$$ (5)

By considering the fact that only *α*_*i*_(*t*) will change in the future, Noori et al. [33] updated Eq (5) so that it can be used for prediction as:

$$DOISSTA(x,t+l)≅\sum_{i=1}^{K}\alpha_{i}(t+l)\Theta_{i}(x)\;K\ll N$$ (6)

where *l* is the future target time.

According to “Future Prediction of SST” in Fig 3, we extrapolated *α*_*i*_(*t*) from 2016 to 2100. Thereafter, the model performance was checked by comparing predicted and measured SST data available from the NOAA website during the control period 2016–2018. Finally, SST data were estimated by application of Eq (6) for PG&GO during 2020–2100. Note that the developed POD model had a spatial and temporal resolution equal to the DOISSTA data used, i.e., 0.25 degree and one day, respectively.

The POD model predicted SST based on the assumption that it will continue to develop according to the pattern embedded in the historical records. Sanford et al. [65] report that emissions of greenhouse gases have been consistent with the representative concentration pathway 8.5 (RCP8.5) of the Fifth Assessment Report of the Intergovernmental Panel on Climate Change (IPCC) [66] from the beginning of 2005. RCP8.5 is similar to emissions scenario A1FI used in the Fourth Assessment Report of the IPCC [67]. Therefore, the POD predictions can be expected to be comparable with those projected by global circulation models such as the Community Model Inter-comparison Project Phase 5 (CMIP5) based on RCP8.5 [68].

## Results and discussion

### Descriptive statistics

**Fig 4:**
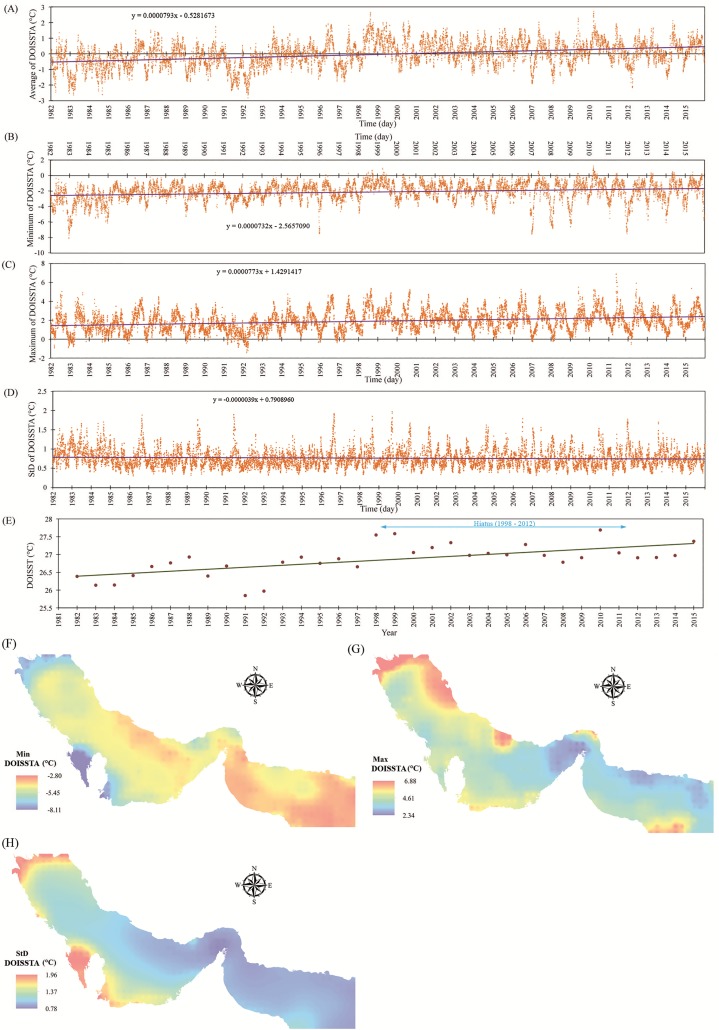
Historical trends in (**A**) mean values, (**B**) minimum values, (**C**) maximum values, and (**D**) standard deviation (StD) values of DOISSTA across the Persian Gulf & Gulf of Oman (PG&GO); (**E**) mean annual values of daily optimum interpolation sea surface temperature (DOISST) across PG&GO; and (**F**) spatial distribution in minimum values, (**G**) maximum values, and (**H**) standard deviation values of DOISSTA across PG&GO.

The mean, minimum, maximum, and standard deviation in DOISSTA for PG&GO (including 609 grids with a spatial resolution of 0.25 degree) are shown in Fig 4A–4D, respectively. As can be seen from Fig 4A–4C, there was an increase in the mean, minimum, and maximum trends of DOISSTA, corresponding to approximately 1 °C over 34 years. This increasing trend in DOISSTA for PG&GO confirms findings by other researchers [21–25,33]. It is worth noting that, even though the increasing trend had a low gradient, it may still be important due to the sensitive marine ecosystem in PG&GO [20,69]. For a better understanding of the variations in DOISST (i.e., not the anomalies) in the study area, mean annual DOISST was calculated for all 609 grids in the region over the 34-year period (Fig 4E). As can be seen from the diagram, there was a gradually increasing trend in mean annual DOISST over the period, with the trend being more pronounced in the beginning of the period (1982–1998) and thereafter relatively stable up to 2015. There was a “hiatus” in the period 1998–2012, due to lower warming rates in ambient air temperature compared with the average warming rate (long-term) and increasing rates of air temperature forecast by climate models [70]. Note that this remains to be confirmed, although it has been detected for both SST and air temperature [70,71]. High DOISST also occurred in 1998–1999, 2002, 2006, 2010, and 2015, years that were subjected to El Niño-Southern Oscillation (ENSO) that eventually led to unusually large DOISST. Some researchers have reported unusually strong and positive DOISST across PG&GO in 1998, 2002, 2006, and 2010, possibly as a result of the Indian Ocean Zonal Mode, which is connected to ENSO [20,22,72]. A link between positive DOISSTA in PG&GO and ENSO status in the Pacific has also reported [41].

The spatial variation in minimum, maximum, and standard deviation of DOISSTA are shown in Fig 4F–4H, respectively. A generally decreasing trend in minimum values from east to west was clearly evident in the study area (Fig 4F). In other words, the Persian Gulf, located in the western part of the study area, has experienced smaller DOISSTA than the Gulf of Oman. The minimum values of DOISSTA occurred along the southern coast of the Persian Gulf, between the Bahrain and Qatar coastlines (Gulf of Salwah), and in the northernmost areas of the Persian Gulf, where the river Arvand Rud discharges into the Persian Gulf. The northernmost areas of the Persian Gulf experienced the maximum values of DOISSTA. These areas are the shallowest parts of the Persian Gulf, where the water temperature is highly affected by the air temperature of the surrounding dry land. Furthermore, the water temperature of Arvand Rud, which differs from that of the Persian Gulf, plays a key role in the fluctuation in SST in the northernmost parts of the gulf. Similar to the minimum values, there was an increasing trend in maximum values of DOISSTA from east to west (Fig 4G). As regards the standard deviation of DOISSTA, it appeared that the majority of the Gulf of Oman experienced the smallest variation, due to its greater water depth and higher latent heat of vaporization (Fig 4H). The central parts of the Persian Gulf and locations close to the Strait of Hormuz experienced low variation in DOISSTA, due to greater water depth compared with other parts of the gulf. The southern coast and northernmost part of the Persian Gulf experienced the highest DOISSTA variations, as a result of being affected by air temperature of the surrounding dry land, shallow water depths in the region, and connections between the Arvand Rud and the Persian Gulf. These findings are comparable with results reported by Nandkeolyar et al. [22].

### POD results

#### Mode extraction

**Fig 5:**
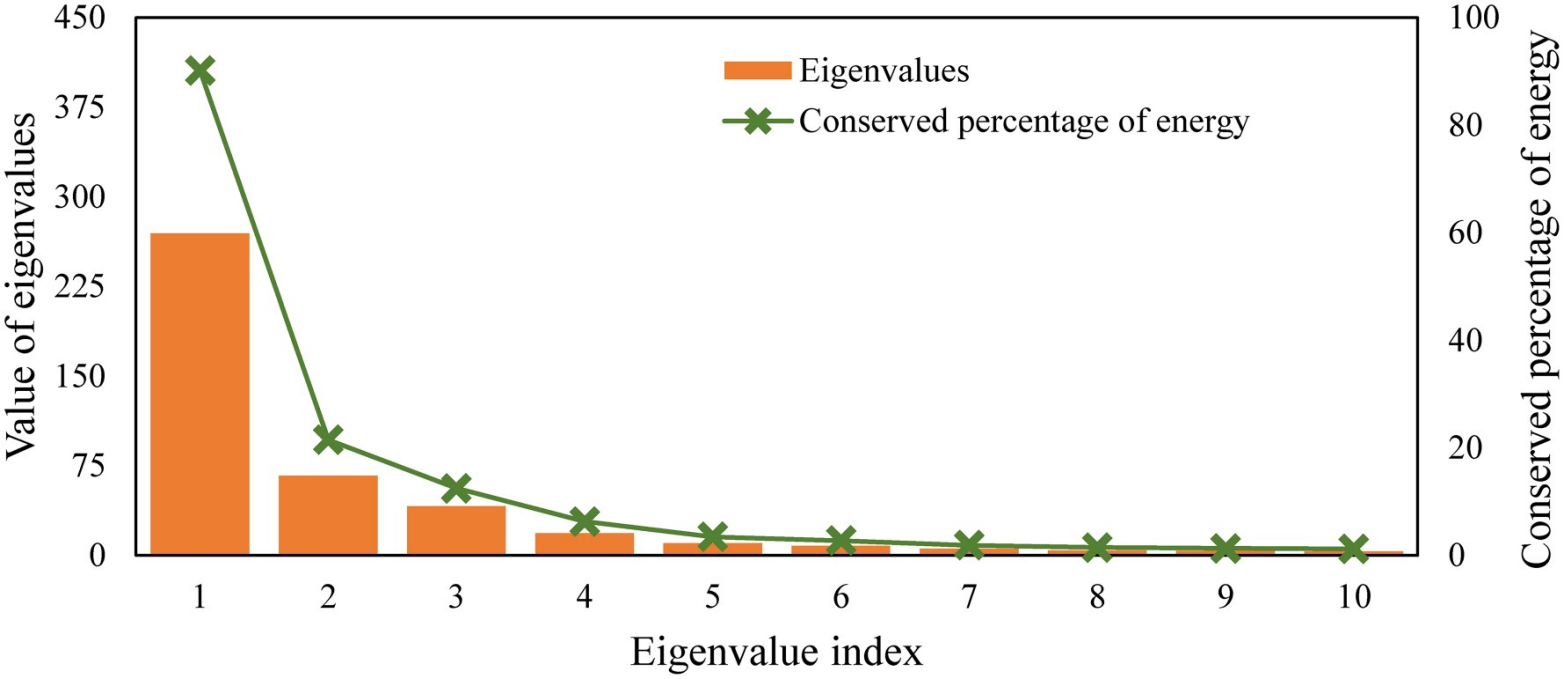
The first 10 calculated eigenvalues and their conserved energy percentage in the Persian Gulf & Gulf of Oman (PG&GO).

The first 10 calculated eigenvalues, along with their conserved system energy, are shown in Fig 5. The first eigenvalue conserved about 60% of the system energy and the first five eigenvalues represented about 90% of total energy. In general, there is no a distinct formula to choose the number of modes corresponding to the eigenvalues for further investigation of an objective parameter. It is usually determined based on an energetic threshold criterion greater than 90% as used by some researchers [56,57,64]. In addition, in this study, just the first five modes have to be included as the other eigenvalues are too small to be of interest (Fig 5). Thus, using the first five modes corresponding to the first five eigenvalues, it was possible to determine the dominant pattern of DOISSTA variation in PG&GO.

**Fig 6:**
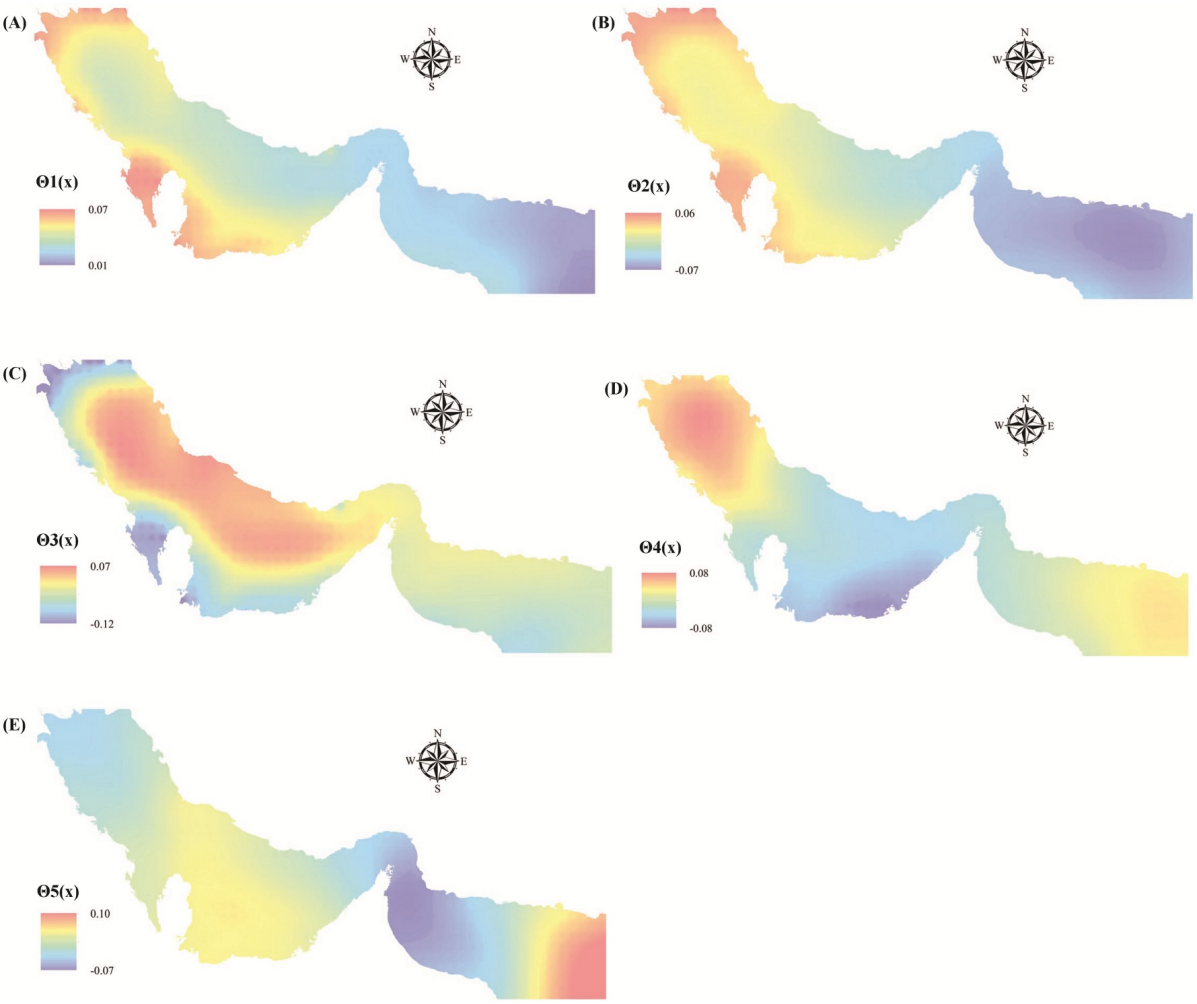
Pattern of space-dependent terms for (**A**) the first mode **Θ**_1_(**x**), (**B**) the second mode **Θ**_2_(**x**), (**C**) the third mode **Θ**_3_(**x**), (**D**) the fourth mode **Θ**_4_(**x**), and (**E**) the fifth mode **Θ**_5_(**x**).

The space-dependent terms for the first five modes (**Θ**_1_(**x**) to **Θ**_5_(**x**), respectively) are shown in Fig 6A–6E, respectively. According to the results for **Θ**_1_(**x**), the Persian Gulf has experienced more variation in DOISSTA than the Gulf of Oman (Fig 6A). In addition, the shallow parts of PG&GO, which are mostly located along the southern coasts and northernmost parts of the Persian Gulf, showed the largest variation. Based on the results for **Θ**_1_(**x**), the Gulf of Oman has experienced the least variation, due to its greater depth than the Persian Gulf. These findings are in line with results reported by Nandkeolyar et al. [22]. Comparing the results of **Θ**_1_(**x**) with the spatial distribution of standard deviation calculated in the previous stage (see Fig 4H) revealed that, although the results were rather similar, there were some differences. These differences were mainly due to consideration of information from all grids in the study area for calculation of **Θ**_1_(**x**), which leads to more realistic results [73]. For example, considering the shallower water depth in the Persian Gulf than in the Gulf of Oman, DOISSTA in the Persian Gulf can be expected to be more affected by the air temperature of surrounding dry land, which displays greater variation. Therefore, **Θ**_1_(**x**) more accurately reflected the actual spatial variation in DOISSTA in PG&GO than the calculated standard deviation in Fig 4H. Moreover, the **Θ**_2_(**x**) results were similar to the results for **Θ**_1_(**x**), especially in the Persian Gulf. For example, based on the **Θ**_2_(**x**) results, there was significant variation in DOISSTA along the southern coast and the northwestern part of the Persian Gulf during the study period (Fig 6B). In addition, the **Θ**_3_(**x**) and **Θ**_4_(**x**) results (shown in Fig 6C and 6D, respectively) support the results for **Θ**_1_(**x**), with both components reflecting higher variation in southern and northernmost parts of the Persian Gulf. However, the results for **Θ**_5_(**x**) showed little compatibility with **Θ**_1_(**x**), as they indicated that coasts along Oman and United Arab Emirates in the Gulf of Oman experienced more variation in DOISSTA (Fig 6E).

**Fig 7:**
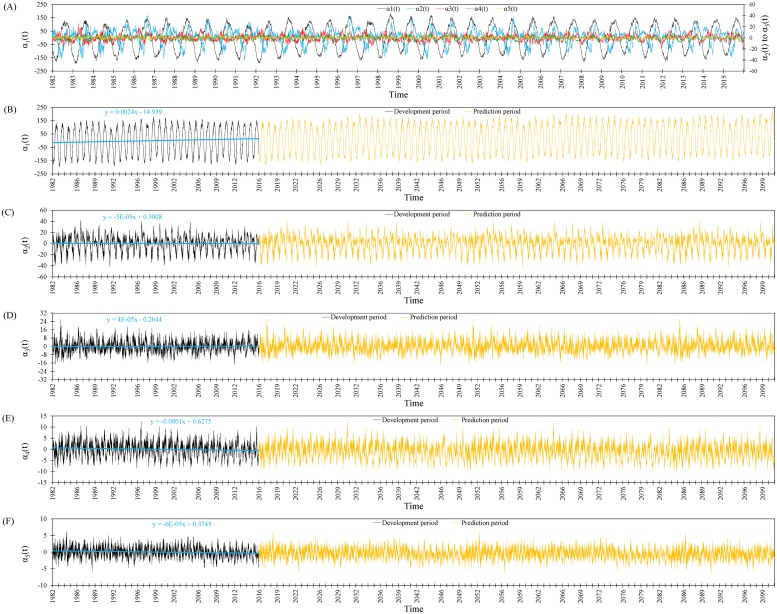
Pattern of time-dependent terms for (**A**) the first mode *α*_1_(*t*), (**B**) the second mode *α*_2_(*t*), (**C**) the third mode *α*_3_(*t*), (**D**) the fourth mode *α*_4_(*t*), and (**E**) the fifth mode *α*_5_(*t*).

The calculated temporal terms of all first five modes (*α*_1_(*t*) to *α*_5_(*t*), respectively) are shown in Fig 7A, while those for *α*_1_(*t*) to *α*_5_(*t*) are shown separately in Fig 7B–7F, respectively. It is worth noting that *α*_1_(*t*) had greater importance than the other terms due to the significance of the first mode (**Θ**_1_(**x**)). As Fig 7A clearly shows, *α*_1_(*t*) to *α*_5_(*t*) fluctuated with an annual wavelength that matched the annual change cycle in SST. According to the results for *α*_1_(*t*) (Fig 7B), the variation in DOISSTA was incremental and increased with a low gradient during the 34-year study period, as also reported by others [21–25,33], and the minimum DOISSTA occurred in late February each year. Thereafter, there was a warming trend, with DOISSTA reaching a maximum by the middle of August, followed by a cooling period, so that DOISSTA again reached a minimum in late February. This cycle was repeated for *α*_1_(*t*) in each year in the study period, and is in line with reported results for SST variation in the study area [17]. Based on the high percentage of system energy conserved by the first mode, the results for *α*_1_(*t*) represented a high percentage (about 60%) of the DOISSTA variation in PG&GO. The results for *α*_2_(*t*), *α*_4_(*t*), and *α*_5_(*t*) indicated a mild decreasing trend for DOISSTA (Fig 7C, 7E and 7F, respectively). These findings clearly suggest a cold bias in DOISSTA across PG&GO, which matches reported results obtained by running climate models [74]. This cold bias in DOISSTA across PG&GO regularly expands in winter and continues into spring and summer [75].

The high variation in DOISSTA across PG&GO compared with the concurrent global average may be a result of different phenomena [21]. El Niño-Southern Oscillation and the Indian Ocean Zonal Mode are undoubtedly two processes that are important drivers of DOISSTA in PG&GO. However, it has been suggested that the interplay between ENSO and the Indian Ocean Zonal Mode is not the only mechanism influencing DOISSTA in PG&GO [41]. Other investigations have indicated that a positive North Atlantic Oscillation, the Siberian high pressure system, and Shamal wind events have important effects on DOISSTA [76,77]. Furthermore, consistent warming trends for PG&GO have been reported since 1950, and especially since 1970 [78]. The air temperature over PG&GO is rapidly increasing and the increase may exceed +4 °C by the end of this century [79]. Furthermore, there is some evidence of a weakening influence of the Siberian high pressure system that could be a result of an increasing trend in air surface temperature over PG&GO [80]. All these results indicate that the positive rate of warming of PG&GO water could be related to the increasing trend in climate variables such as air temperature in the study area, as well as strong effects of oceanographic events such as ENSO, the North Atlantic Oscillation, and the Indian Ocean Zonal Mode.

#### Model performance for control period 2016–2018

**Fig 8:**
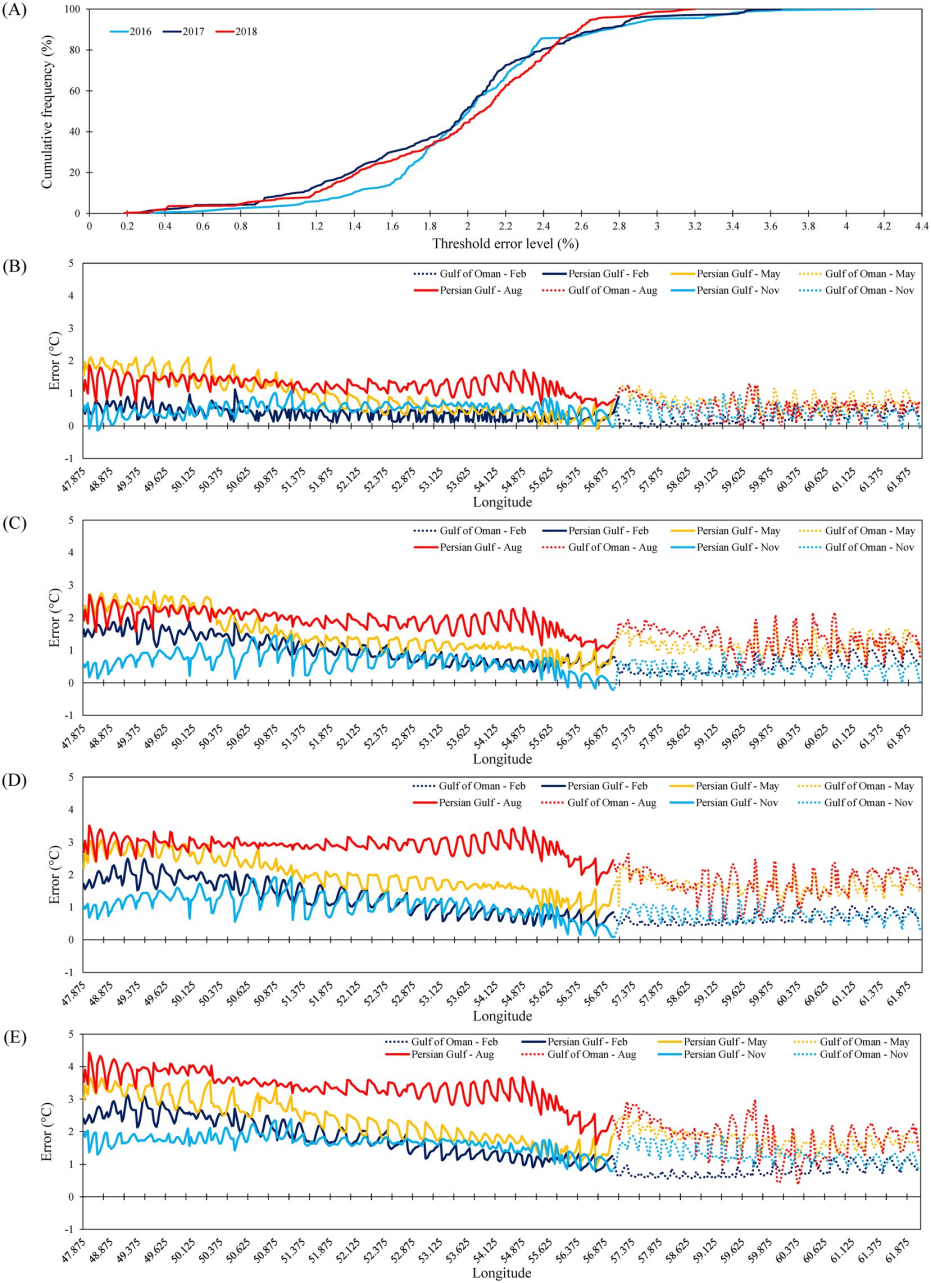
(**A**) Mean absolute relative error between sea surface temperature (SST) values for the Persian Gulf & Gulf of Oman (PG&GO) predicted by the proper orthogonal decomposition (POD) model and measured during the period 2016–2018; and spatial trends in mean monthly SST in (**B**) February (representing winter), (**C**) May (representing spring), (**D**) August (representing summer), and (**E**) November (representing fall) in the selected future years 2030, 2050, 2070, and 2100.

The *α*_1_(*t*) to *α*_5_(*t*) values for the period 2016–2100 estimated using the POD model are shown in Fig 7B to 7F, respectively (extrapolated values are shown in brown). The model performance was checked by comparing predicted and measured SST data available from the NOAA website for the control period 2016–2018. The mean absolute relative error between SST values predicted by the POD model and measured values for 2016–2018 is shown in Fig 8A. The error was less than 2% for more than 50% of days in 2016, 2017, and 2018, while it increased to about 2.7% for more than 90% of days in 2016, 2017, and 2018 (Fig 8A). Based on these results, it can be concluded that the POD model reasonably predicted SST for PG&GO.

#### Predictions: 2020–2100

**Fig 9:**
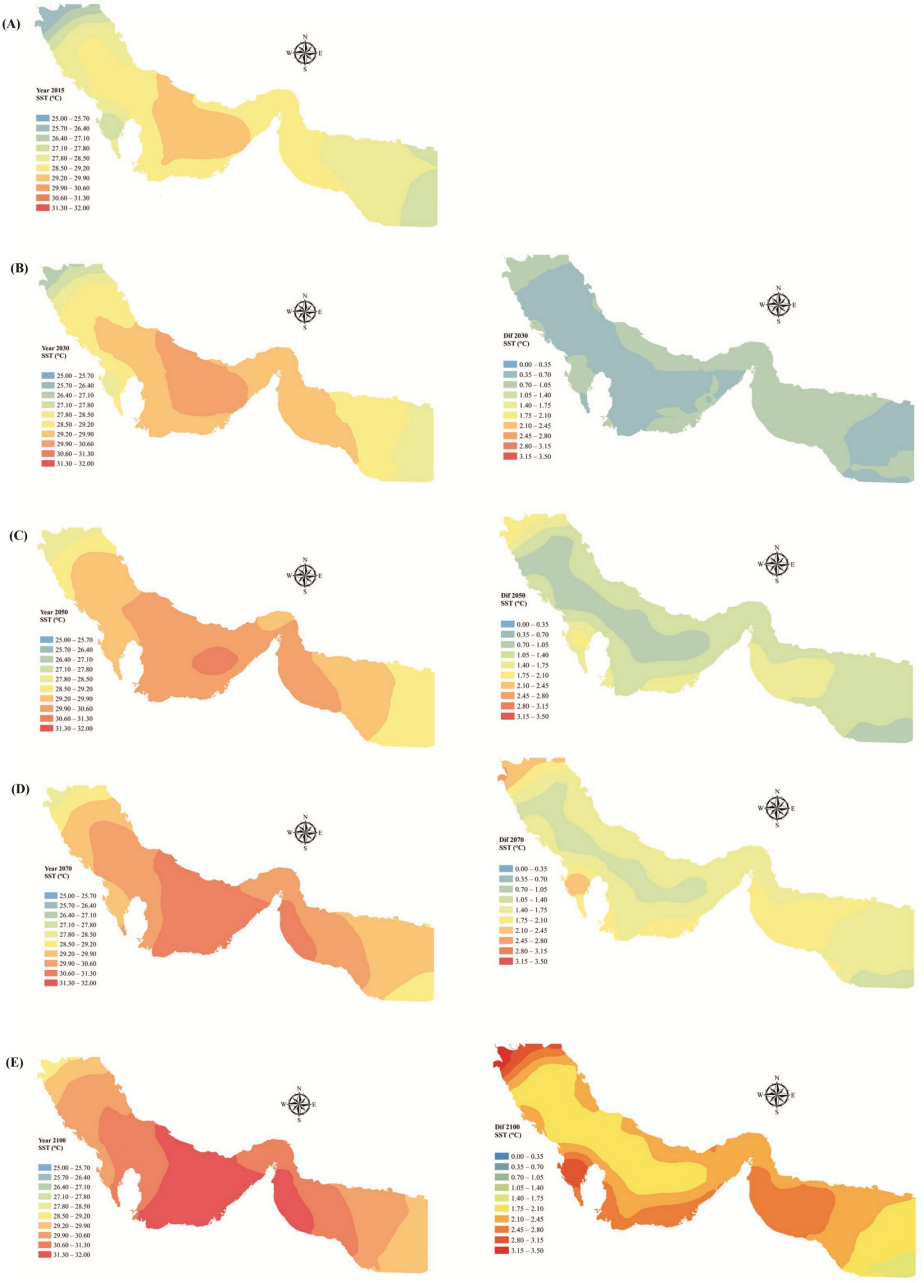
(**A**) Spatial distribution of mean annual sea surface temperature (SST) across the Persian Gulf & Gulf of Oman (PG&GO) for (**A**) the year 2015, (**B**) the year 2030 and difference between 2030 and 2015, (**C**) the year 2050 and difference between 2050 and 2015, (**D**) the year 2070 and difference between 2070 and 2015, and (**E**) the year 2100 and difference between 2100 and 2015.

The POD model was used to predict SST across PG&GO for the period 2020–2100. Spatial trends in mean monthly SST in February, May, August, and November (representing winter, spring, summer, and fall respectively), are illustrated in Fig 8B–8E for the selected years 2030, 2050, 2070, and 2100, respectively. The spatial distribution of mean annual SST for the selected years 2015, 2030, 2050, 2070, and 2100 is shown in Fig 9A–9E, respectively. In addition, the spatial distribution of differences between the predicted SST shown in these diagrams and those measured in 2015 are illustrated in Fig 9B–9E. All diagrams clearly show a warming trend in PG&GO in the future, and a more severe warming trend in the Persian Gulf than in the Gulf of Oman.

Based on Fig 8B–8E, summer seasons in the Persian Gulf will experience most warming by 2100, while fall seasons will experience least. Summer seasons in the Gulf of Oman will also experience most warming by 2100, while winter seasons will experience the least warming in that case. In general, the maximum difference between SST measured in 2015 and projected by the POD model was observed for the Persian Gulf, where SST was 4.3 °C higher by August 2100. The greatest differences for the Persian Gulf in other future years were 2.1, 2.8, and 3.4 °C in May 2030, May 2050, and August 2070, respectively. No previous study has projected SST for PG&GO by the end of 21^st^ century, but the results obtained are in line with values reported for other marine environments. For example, the monthly SST projected by global circulation models under RCP8.5 indicates a rise of 4.8 and 3.7 °C for six large marine ecosystems of the Northwest Atlantic by the end of August and February 2100, respectively [81]. Results from 26 models in CMIP5 under scenario RCP8.5 reveal an increase of up to 6 °C from August 1976 to August 2099 for monthly SST over large marine ecosystems adjacent to Europe, North America, and the Arctic Ocean [28]. Additionally, SST over the Mediterranean Sea during summer and winter seasons has been estimated to rise by 2.9 and 0.5 °C, respectively, under scenario RCP8.5 by the end of 21^st^ century [44].

In regards to mean annual warming, this study clearly showed that the north coast of the United Arab Emirates and south coastlines of the Strait of Hormuz will experience the highest SST in the future, reaching 32 °C by 2100 (Fig 9B–9E). Mean annual (maximum) SST across PG&GO may increase from 28.5 (29.9) °C in 2015 to 30.7 (31.8) °C in 2100. Such an increase would threaten natural habitats in PG&GO [82]. The increasing trend in the Mediterranean Sea is reported to about 3.1 °C under scenario A2 (same as A1FI, represents the highest emissions of greenhouse gases) for the period 1960–2099 [29]. The SST off the Australian coast is predicted to increase by 2–4 °C under RCP8.5 by 2100 [83]. Chust et al. [84] predict a positive change in SST under scenario A1B (represents moderate emissions of greenhouse gases) over the Black Sea, of around 3.7 °C during 2080–2100 relative to 1980–2000. The increase in annual SST for PG&GO predicted in the present study also exceeded the up to 1.3 °C increase in global SST during 21^st^ century predicted by Aral and Guan [31]. In another study, the SST in the Western English Channel was predicted to increase by around 0.5 and 2.5 °C under scenarios RCP2.6 and RCP8.5, respectively, by the year 2100 [85].

Relative to the year 2015, our predicted monthly maximum SST warming in PG&GO by the end of 21^st^ century is close to that estimated for large marine ecosystems and exceeds that estimated for the Mediterranean Sea. In addition, we found that summers generally showed a stronger trend than winters. This may be a result of integration of greenhouse gas warming over a much thinner climatological mixing layer depth in warm (summer) than in cold (winter) seasons, thereby intensifying the SST seasonal cycle during the 21^st^ century [28].

## Conclusions

This study evaluated variations in DOISSTA across PG&GO during the past 34 years using descriptive statistics and predicted SST by 2100 using a POD model. Investigations of DOISSTA based on statistical indices revealed an increasing trend in mean, minimum, and maximum DOISSTA of about 1 °C during the 34-year study period, likely due to the impacts of climate change. In the study period, the Persian Gulf experienced smaller and larger DOISSTA than the Gulf of Oman. The spatial distribution of the first mode calculated by POD revealed maximum variation in DOISSTA in the Persian Gulf, especially along its southern coasts between Bahrain and Qatar coastlines and in the northernmost area, where the river Arvand Rud discharges into the gulf.

Prediction of SST indicated that summer seasons in PG&GO will experience most warming by 2100, while the warming effect will be smaller for fall and winter seasons. The monthly maximum difference between SST measured in 2015 and SST predicted by the model for 2030, 2050, 2070, and 2100 was 2.1 (May), 2.8 (May), 3.4 (August), and (August) 4.3 °C, respectively. Future mean annual warming in PG&GO relative to 2015 was predicted to be highest along the north coast of the United Arab Emirates and south coastlines of the Strait of Hormuz, where SST may reach 32 °C by 2100. Mean annual (maximum) SST across PG&GO was predicted to increase from 28.5 (29.9) in 2015 to 30.7 (31.8) °C in 2100. This 2.2 °C increase in mean annual SST would threaten natural habitats and marine ecosystems in PG&GO.
